# Efficient CPP-mediated Cre protein delivery to developing and adult CNS tissues

**DOI:** 10.1186/1472-6750-9-40

**Published:** 2009-04-24

**Authors:** Yorick Gitton, Lorenzo Tibaldi, Edmond Dupont, Giovanni Levi, Alain Joliot

**Affiliations:** 1 Evolution des Régulations Endocriniennes, CNRS UMR 7221, Muséum National d'Histoire Naturelle, 7 rue Cuvier,75005 Paris, France; 2 Groupe de Biologie Cellulaire des Homéoprotéines, CNRS UMR 8542, Ecole Normale Supérieure, 46 rue d'Ulm, 75005 Paris, France; 3 Current address: Collège de France, Ecole Normale Supérieure, CNRS UMR 8542. 11 place Marcelin Berthelot, 75231 Paris Cedex 05, France

## Abstract

**Background:**

Understanding and manipulating gene function in physiological conditions is a major objective for both fundamental and applied research. In contrast to other experimental settings, which use either purely genetic or gene delivery (viral or non-viral) strategies, we report here a strategy based on direct protein delivery to central nervous system (CNS) tissues. We fused Cre recombinase with cell-penetrating peptides and analyzed the intracellular biological activity of the resulting chimerical proteins when delivered into cells endowed with Cre-mediated reporter gene expression.

**Results:**

We show that active Cre enzymatic conjugates are readily internalized and exert their enzymatic activity in the nucleus of adherent cultured cells. We then evaluated this strategy in organotypic cultures of neural tissue explants derived from reporter mice carrying reporter "floxed" alleles. The efficacy of two protocols was compared on explants, either by direct addition of an overlying drop of protein conjugate or by implantation of conjugate-coated beads. In both cases, delivery of Cre recombinase resulted in genomic recombination that, with the bead protocol, was restricted to discrete areas of embryonic and adult neural tissues. Furthermore, delivery to adult brain tissue resulted in the transduction of mature postmitotic populations of neurons.

**Conclusion:**

We provide tools for the spatially restricted genetic modification of cells in explant culture. This strategy allows to study lineage, migration, differentiation and death of neural cells. As a proof-of-concept applied to CNS tissue, direct delivery of Cre recombinase enabled the selective elimination of an interneuron subpopulation of the spinal cord, thereby providing a model to study early events of neurodegenerative processes. Thus our work opens new perspectives for both fundamental and applied cell targeting protocols using proteic cargoes which need to retain full bioactivity upon internalisation, as illustrated here with the oligomeric Cre recombinase.

## Background

From focal demyelination or axonal degeneration events characterizing peripheral neuropathies, to localized genetic alterations involved in primary neuro-oncogenic processes, understanding cell- and domain-specific gene function in the CNS [1-4] often requires the modification of gene expression in restricted cell populations. Cre-mediated recombination has become a key strategy for the spatial and temporal control of gene inactivation or over-expression. This has been mainly achieved through genetic approaches, by crossing transgenic mouse strains carrying loxP-flanked ("floxed") genes with transgenic mice expressing the Cre recombinase under the control of specific promoters, or through viral or non-viral delivery of Cre encoding DNA. With the latter strategy, spatial restriction of the infected tissue to defined areas remains challenging. As an alternative strategy, direct delivery of therapeutic agents into cells ('transduction'), has had numerous and encouraging attempts [5-10]. Endogenous peptides endowed with internalisation properties (Cell Penetrating-Peptides, hereafter termed "CPPs") have strongly improved the efficiency of delivery [11]. Fragments of TAT [10,12], FGF [13], and homeoproteins are efficient peptidic vectors, which do not have obvious deleterious impact on cell survival; they have been combined, either chemically or genetically, with different macromolecular cargoes [5-9]. However, the restricted delivery of such compounds in topographically focalized territories has not been directly evaluated in the case of neural cells, which are highly heterogeneous in term of spatial identity. We have addressed this issue with the intra-cellular delivery of Cre recombinase fused to CPPs, a strategy which has been already well described [13-18]. Cre recombinase is a large hydrophilic enzyme frequently used to perform targeted genomic recombination [19], resulting in permanent reporter transgene expression when using dedicated genetic backgrounds. This functional read-out unambiguously ensures the presence of a biologically active Cre protein inside the nucleus, contrasting with the visualisation of protein internalization that has lead to conflicting results [20-25]. We show the efficient delivery of the active enzyme in topologically restricted cell populations and provide several examples of the applications of this technology to neuronal targeting and cancer research, a strategy which obviates the need for sustained expression of Cre recombinase [26-29] through genetic procedures.

## Results

### Fusion to Penetratin-based CPPs preserves recombinase activity and promotes nuclear Cre internalization

**Figure 1:**
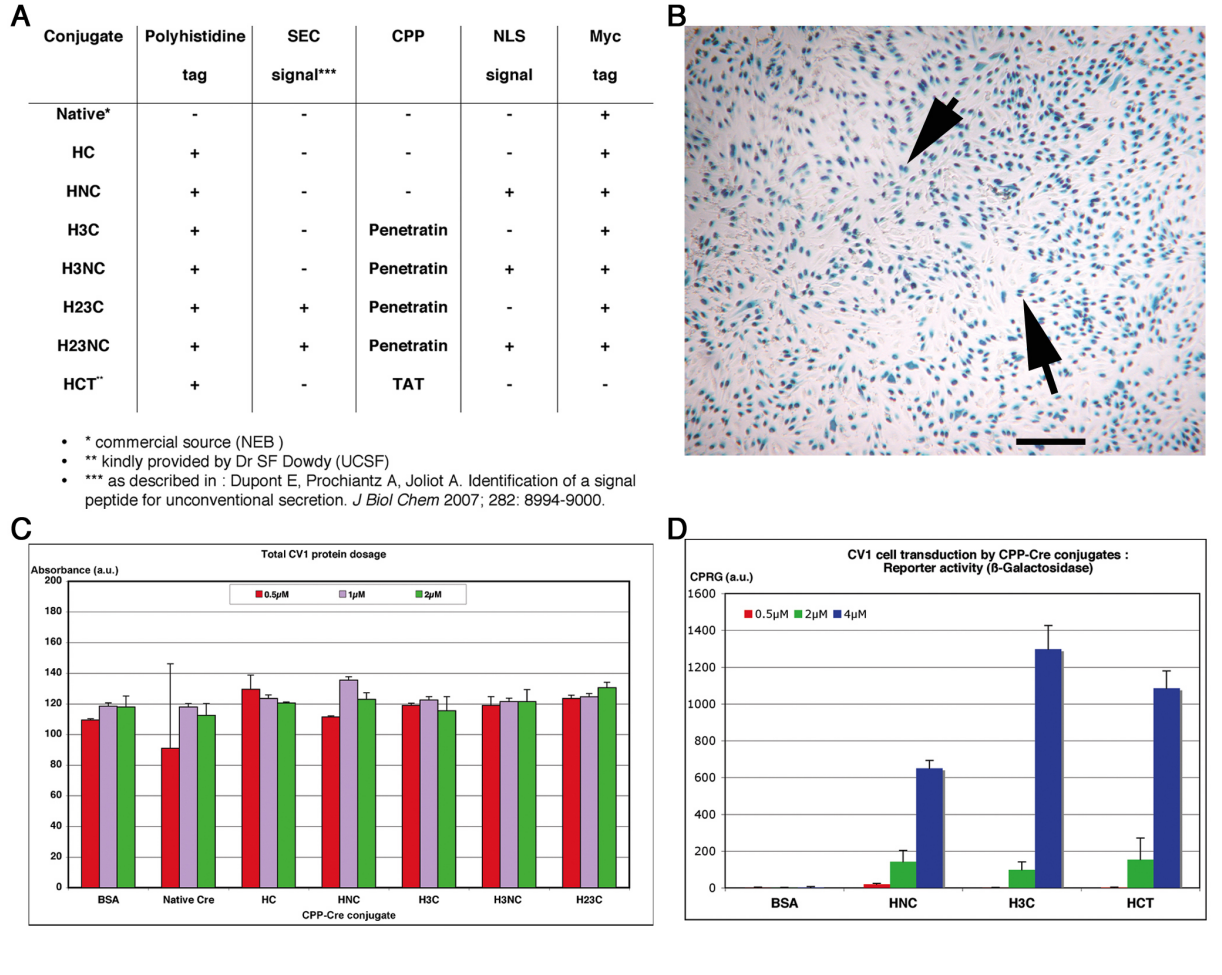
**Genomic recombination upon transduction of CPP-Cre conjugates in reporter cells**. (A) Peptidic additions to Cre recombinase: 'NLS', nuclear localization signal; '3' or 'Penetratin', Antennapedia transduction domain, see [5]; '23' or 'SEC', Antennapedia secretion signal, see [33]; 'TAT', transduction domain from HIV Tat protein (see [10]); 'myc', myc tag; 'his', polyhistidine tag. See also Additional file 1. (B) β-Galactosidase activity (arrow) of CV-1B reporter cells following 1 h incubation with H3C. Arrows, pairs of daughter cells from individually transduced cells. (C) Bradford quantification of total protein content after conjugate exposure showed unaffected cellular survival upon transduction. MTT cell viability assays on the same samples indicated that conjugate doses only above 5 μM were cytotoxic (not shown). (D) Dose-dependent transduction assay. Reporter activity was quantified 48 h after 1 h exposure to conjugates. The indicated proteins showed strongest activity at 4 μM, with a maximum for H3C. H3NC reached similar levels (see Additional file 2D). Scale bar in B represents 1800 μm.

Cre recombinase coding sequence was genetically inserted in frame with several peptidic coding sequences coding for CPPs and the recombinant proteins were purified from bacteria (Fig. 1A & Additional file 1A). Enzymatic activity was monitored in cell-free conditions, through recombination of a target linear plasmid harbouring two *loxP *sites. All fusion proteins displayed similar specific recombination activities, in the range of 4 units/μg – except for Tat-Cre, which was significantly more active (16 units/μg), outperforming a commercial Cre (NEB #MO298, 11 units/μg; see Additional file 1B). As both the nuclear localization signal (NLS) and the highly basic character of the Tat and Penetratin sequences might have enhanced Cre biological activity by increasing its nuclear accumulation, independently of the internalization process [30,31], the recombination activities of histidine-tagged Cre conjugates (HC, hNC and H3C) were further evaluated by transient transfection. The three coding sequences, sub-cloned into a mammalian expression plasmid, were expressed in CV1B cells, which display β-Galactosidase reporter activity upon Cre-mediated recombination [32]. Cre immunodetection indicated that all proteins displayed similar expression and nuclear accumulation levels (see Additional file 2A–C). Furthermore, detection of reporter activity showed similar target recombination efficiency for all tested Cre conjugates, consistent with previous observations [30,31]. This efficacy further applied to the Antp secretion signal-appended Penetratin conjugates (H23C and H23NC), suggesting a robust nuclear targeting by the Penetratin moiety [33]. These results rule out nuclear addressing as a limiting step in Cre nuclear activity during protein transduction assays – which thus allows correlating their transduction efficiencies with the internalization step.

We then evaluated the biological activity of these proteins upon transduction of the adherent CV1B reporter cell line [32]. Fusion of HC to Tat, NLS or Penetratin, resulted in equivalent recombination efficiency and cell survival (Fig. 1B–D) upon exposure for 60 mn. Maximal activity was obtained at 4 μM, resulting in 80% of β-Galactosidase positive cells (Fig. 1D). By contrast, the native HC protein displayed a low but significant transduction rate, with less than 20% of reporter-expressing cells. Interestingly, as previously reported, the transduction efficiency of NLS, which is not considered as a bona fide CPP, was twice that of Cre alone [32]. The simultaneous addition of both NLS and Penetratin sequences to Cre protein (H3NC) only slightly enhanced the transduction efficiency compared to H3C (see Additional file 2D). Reducing the exposure of cells to H3NC (4 μM) from 60 down to 15 mn resulted in recombination efficiency levels close to maximum (see Additional file 2E), suggesting a fast interaction of the protein with the cell surface.

### CPP-Cre fusion proteins as a new tool for the study of neural development

**Figure 2:**
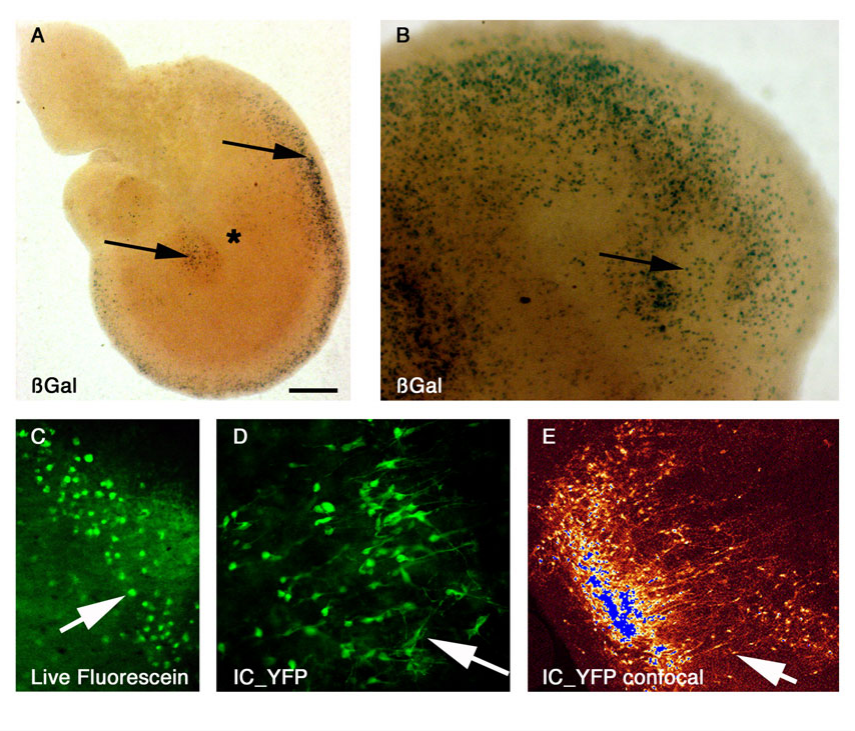
**CNS explants from Cre-dependent transgenic embryos displayed reporter activity upon exposure to CPP-Cre fusion proteins**. Telencephalic explants of dissected fragments from the embryonic brain (see Additional file 3), were obtained and cultured as previously described, then exposed to protein conjugates added at the center of the explant (asterisk). Two to ten days later, transgene-expressing cells (arrows) were detected by reporter enzymatic activity (A, B), intrinsic fluorescence (C), or immunohistochemistry (D-E). Vehicle-treated explants were devoid of reporter expressing cells (see Additional file 4). (A, B) Explants (R26R strain) exposed for 96 h to a 1 μl drop of 100 ng/μl H3NC. Visualization of reporter activity (X-gal staining; positive blue cells indicated by arrows) showed reporter-expressing cells, located mainly in the peripheral domain (B). (C) Explant from RYFP strain, exposed for 8 h to a 1 μl drop of 100 ng/μl H3NC. Live fluorescence recording (GFP channel; bright green cells, arrow) shows early recombinant cells in the centre of the explant. (D-E), YFP immunofluorescence analysis using a pan-GFP antibody revealed reporter expression in large cell clusters, 72 h after H3NC transduction (1 μl drop of 100 ng/μl). In (D), labelled cells were classically documented while in (E), reporter YFP was analysed using confocal analysis (pseudo-colors GLOW-LUT converted representing signal intensity, blue indicating saturated signal). In A-E, arrows point to representative recombinant cells, including those with a morphology suggestive of neuronal identity in pictures D-E. For the whole figure, the scale bar in A represents: A, 350 μm; B, 750 μm; C, 150 μm; D, 50 μm; E, 1000 μm.

**Figure 3:**
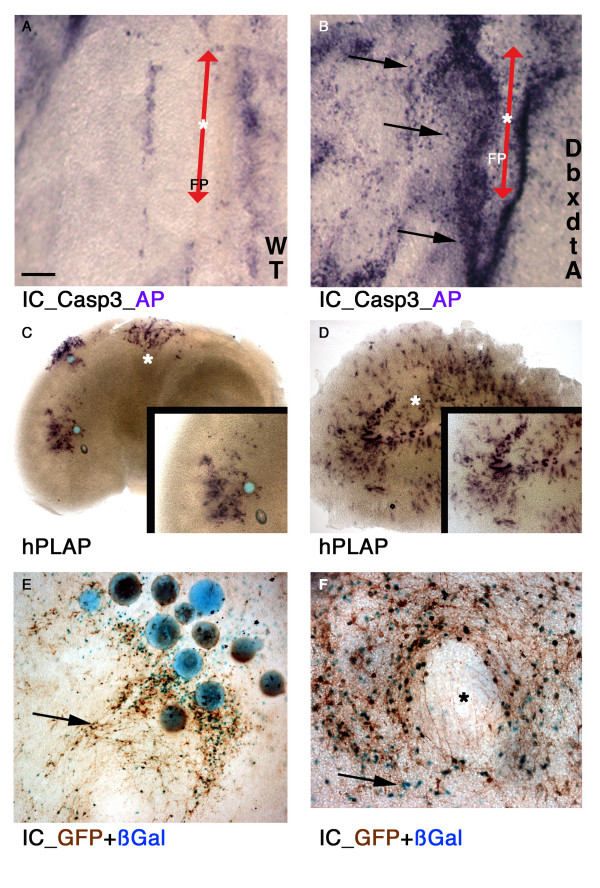
**Focal recombination obtained through genetic and mechanical approaches**. (A, B) Genetic approach. Conditional induction of a caspase-dependent pro-apoptotic program in a neuronal subpopulation. Flat-mount embryonic spinal cord explants, from wild-type (A) or *Dbx1-DTA *strain (B), exposed for 96 h to 1 μl of 400 ng/μl H3NC. Activated-caspase III immunodetection showed induction of early steps of apoptosis only in transgenic explants exposed to the conjugate (B; arrows: representative positive cells, purple staining). The apoptotic marker was not induced in non-transgenic tissue (A). Likewise, vehicle solution did not induce significant apoptosis (see Additional file 4C). Red double arrow: floorplate (see also Additional file 3). (C-F) Mechanical approach. Focal conjugate delivery produced clusters of recombined cells. (C) H3NC-soaked beads (200 ng/μl) were deposited onto Z/AP cortical explants. hPLAP reporter activity was monitored 24 h later (purple precipitate). Inset, clusters of radiating positive cells. (D) In contrast, the drop assay using the same protein solution (200 ng/μl) on parallel explants led to widely dispersed reporter-expressing cells (inset, higher magnification). (E-F) Combination of genetic and focalized restriction of cell targeting. Detection of simultaneous reporters (β-Galactosidase, blue; GFP, brown; arrow) induced by H3NC (200 ng/μl) in TGZ cortical explants. In both C (top cluster of positive cells, asterisk) and F explants (center, asterisk), one bead was removed, exposing cells in contact with the beads. Arrows (E, F): representative nuclei surrounded by dense axon bundles, suggestive of neurons. For the whole figure, scale bar in A represents: A, B, E, 300 μm; C, D, 1000 μm; F, 150 μm.

**Figure 4:**
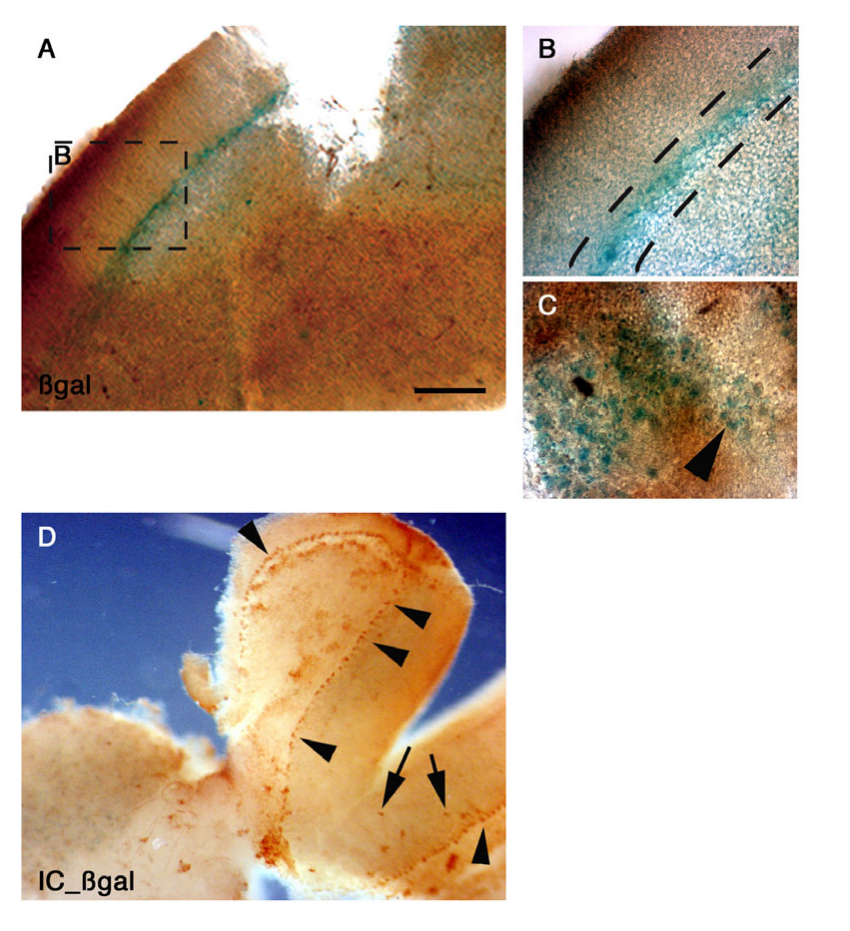
**Adult tissues are amenable to topographically restricted cell targeting**. (A-C) Adult forebrain slice explants (R26R strain), exposed to a 1 μl drop of 100 ng/μl H3NC, displayed reporter β-Galactosidase activity (blue staining, arrows) throughout brain regions. Boxed area in A is observed at higher magnification in B. Dashed lines in B encompass periventricular layers. (C), higher magnification in the striatum. The arrow points to representative recombinant cells arranged as glomerula. (D) Fragment of adult cerebellum explants (TGZ strain) exposed to H3NC (100 ng/μl), displayed reporter alkaline phosphatase immunopositive cells, most of which appear arranged in a laminar organization suggestive of Purkinje cell layer. The arrowheads point to representative recombinant cells within the Purkinje cell layer, while the arrows indicate rare positive cells outside of it. For the whole figure, the scale bar in A represents: A, 1600 μm; B, 800 μm; C, 130 μm; D, 3000 μm.

We next addressed whether CPP-Cre mediated recombination would be suitable for the study of neural tissue. In preliminary experiments we had observed that primary neuronal cell cultures were readily amenable to transduction (data not shown). To gain further insight into the efficiency of transduction at the tissular level, we developed transduction assays on explants [34-36] of adult or embryonic CNS regions derived from Cre-inducible reporter transgenic mice. The explants were cultured in serum-free medium and subsequently exposed to CPP-Cre fusion proteins (see Additional file 3). Tissue fragments were overlaid with a 1 μl drop of protein solution (10 to 400 ng/μl) and assayed for reporter activity, 1 up to 10 days later. None of the explants from reporter strains used in this study displayed recombination events upon exposure to vehicle solution (see Additional file 4). Firstly, drops of CPP-Cre were applied at the centre of telencephalic explants from E12.5–E16.5 mouse embryos (Fig. 2 and Additional file 5). Dose-response assays showed that exposure to most of these conjugates resulted in a steep response, with no or barely detectable recombination events below 100 ng/μl, and maximal reporter expression from 200 ng/μl with all tested transgenic reporter strains (see Additional files 5 and 6). Since all tested CPP or NLS conjugates induced recombination with similar efficiencies, only tissues exposed to H3NC were hereafter used to illustrate representative assays in Fig. 2, 3 and 4. As documented in Fig. 2A, B, reporter-expressing cells from R26R-derived explants were detected throughout the explant from the 200 ng/μl concentration on (see also Additional file 6). In contrast, conjugate titres below this limit resulted in restricted clusters of targeted cells (see Additional file 6). Within live cortical explants from transgenic mice carrying fluorescent inducible reporters, gene expression was detected as soon as eight hours after transduction by direct fluorescence (Fig. 2C). Indirect immunodetection and confocal analysis (Fig. 2D–G) performed 72 h later suggested that neurons might be preferential targets for CPP-Cre internalization, based on the morphology of the expressing cells (Fig. 2D, E) – consistent with previous observations [37].

Although indicative of efficient recombination, the drop delivery approach resulted in a heterogeneous distribution of reporter-expressing cells, either at the centre or at the periphery of the explant, which could reflect the persistent drift of the proteins towards the edge of the explants. We thus examined whether recombination could be further restricted to cell subpopulations of the developing CNS thanks to the use of lineage-specific promoters. For this purpose we targeted a single lineage of neuroblasts in a model of neuronal apoptosis. The fusion proteins were used to transduce E9.5–E14.5 spinal cord explants from the *Dbx1-Dta *strain (kindly provided by Dr A. Pierani [38,39]), in which only *Dbx1*-positive interneurons express the pro-apoptotic diphtheria toxin A fragment. Apoptosis can then be evidenced by induction of activated caspase III. Upon transduction, apoptotic cells could be detected only within transgenic explants exposed to Cre fusion proteins (Fig. 3B) but not with vehicle solution (see Additional file 4). These cells were arranged along two parasagittal lines, consistent with the distribution of *Dbx1 *positive interneurons (A. Pierani, personal communication). In contrast, fusion proteins did not induce significant apoptosis in non-transgenic explant (Fig. 3A). Expression of the target transgene, performed here to monitor early events during cell death, can thus be induced in a lineage-restricted population, depending upon the activity of its promoter. Furthermore, these observations underline the innocuous character of the exposure to CPP-Cre conjugates, as illustrated by the absence of significant apoptosis in *Dbx1 *negative cells.

Next we asked whether topologically restricted recombination could be obtained using ubiquitously expressed instead of cell-type restricted reporter transgenes. To spatially restrict recombination, we focused the territory of protein delivery using neutral beads soaked with CPP-Cre proteins. The beads were implanted onto embryonic brain explants derived from the Z/AP strain, in which alkaline phosphatase is expressed upon Cre-mediated recombination. As illustrated in Fig. 3C, cohorts of cells were detected only in close proximity of the beads (inset). In contrast, in a parallel drop assay using the same protein solution used for bead soaking, positive cells were widely dispersed throughout the explant surface (Fig. 3D). The limited diffusion of the fusion proteins using focal bead implantation thus allows spatial targeting of small cell clusters.

In order to verify that neuronal populations were actually transduced using this protocol, explants from the axon-specific TGZ transgenic strain were incubated with CPP-Cre soaked beads. Following incubation, numerous reporter-expressing neuronal cells characterized by β-Galactosidase positive nuclei and MARCKS-eGFP positive neurites were detected at the vicinity of the beads (Fig. 3E, F). This assay further illustrates the modularity of the system, with the restriction of reporter expressing cells by a combination of focal delivery and promoter-specific transgenic lines.

Whether cells from adult tissues would be similarly amenable to CPP-mediated transduction of Cre recombinase remained to be determined. Serial sections from adult brains of the same mouse strains were exposed to drops of CPP-Cre conjugates. A robust staining developed, delineating the laminar organization of the brain as illustrated in Fig. 4. Noticeably, all Penetratin-based conjugates displayed similar targeting efficiency with the R26R strain, such that transgene-expressing cells spanned the whole surface and thickness of all exposed explants (see Additional File 5). A higher concentration of recombinant cells could be detected in cell-dense domains such as progenitor-rich periventricular layers (Fig. 4A, B) and striatal clusters (Fig. 4A, C). Remarkably, when cerebellar slices were exposed to the drop transduction assay, the predominant reporter-expressing population was constituted by Purkinje cells (Fig. 3D; see [14]). Transduction thus also occurred in mature neurons of the adult CNS.

## Discussion

We recently reviewed [11] the encouraging properties of CPP-based conjugates, including their neural tropism. Efficient direct protein delivery into neural nuclei would greatly improve fundamental and clinical research in neuroscience. Here, we developed non-viral transduction assays based on organotypic cultures from transgenic mice as a surrogate model of live CNS tissue. As reported by others, including *in vivo *studies [8,18,31], we confirm that fusion proteins between CPPs and a large enzyme, the Cre recombinase, are readily translocated through cell membranes and further shuttled inside the nucleus to target genomic loci. Since successful delivery in the CNS of other kind of proteins fused to CPPs have been reported *in vivo *[8,11,40,41], our strategy might thus be not limited to Cre conjugates.

Our study further expands the applications of this strategy to neural explant culture. High levels of recombination were reached on explants upon exposure with as few as 200 ng of protein, and transduction resulted in a mosaic of recombined and unaffected cells within live tissues. Although easy to perform and efficient, the drop approach is poorly reproducible in terms of spatial distribution, mainly because the diffusion of the protein depends on the shape of the explant. With the bead protocol, spatial restriction of recombination to defined areas can easily be achieved, depending upon the position of the bead. In the explant assays, most of the Cre conjugates displayed similar biological activity (see Additional file 5). We only noticed enhanced specific activity of Tat-Cre for cell-free DNA recombination, compared to that of Penetratin-based conjugates (4-fold) – although their cellular activities were similar. This might reveal differential properties of these two CPPs, at least in the context of fusion with Cre protein.

Direct delivery of Cre conjugates presents several advantages over viral strategies, although these latter have been successfully used. In addition to the technical and safety limitations inherent to the production of viruses [42,43], viral-mediated Cre delivery results in the prolonged expression of the enzyme, with possible adverse effects [16,27]. With our approach, we did not observe a dramatic increase in the number of recombinant cells between 8 h and 96 h following treatment (compare Fig 3C, and 3D–E), suggesting the temporal restriction of the recombination events to the first hours of incubation. Indeed, a similar situation is observed in cell culture, where optimal recombination activity is almost reached after a 15 mn exposure to Cre conjugates (see Additional file 2E). Among credible possibilities, the limited stability of the protein in the extracellular medium might account for this observation. This strategy might be used in a pulse application for selecting Cre-dependent genomic constructs or removing undesirable entities from manipulated stem cells, as performed in [16,18].

On cerebral explants, neurons were prime targets for transduction, both upon generalized (drop) and focalized (bead) delivery. In terms of ontogenesis, we and others previously showed that regionalized properties develop early during mouse CNS formation [44-46], resulting from the combined activities of recently identified genetic programs. Which mechanisms underlie neurogenesis might be best understood through focal manipulation of gene function in defined territories. Interestingly, the function of both proliferating and mature post-mitotic neurons of the adult brain can also be approached with this strategy, although the efficiency of the transduction seems decreased.

In adult cerebellar explants, the preferential restriction of recombination events to Purkinje neurons was unexpected and might rely on their larger membrane area offered by their extensive dendritic arborization tree. Whichever mechanism is involved, targeting of this cell type – the largest cell and sole axonal output to extra-cerebellar targets – would be a decisive asset to understand normal and pathologic events during the formation and function of the cerebellum. Peptide-mediated non-viral transfer of proteic cargoes could thus offer a valuable alternative strategy to viral-mediated gene transfer using Purkinje cell-specific targeting elements [29], with the advantage of a non-toxic and rapidly cleared vector [27].

## Conclusion

Controlled inactivation or over-expression of genes both at the spatial and temporal level has become a key strategy in the study of gene function. Our study provides an alternative and complementary strategy to the use of Cre expressing mouse strains or of viral-mediated delivery that can be applied to both explant culture and *in vivo *models. The originality of this strategy relies on the spatial and temporal restriction of recombinant cells without the need of specific promoters. We demonstrate that this method of transduction could find innovative applications in different areas of neuroscience including neural development and neurophysiology.

## Methods

### Expression vectors of CPP-Cre fusion proteins

Plasmids were derived from pTHCremyc and pTHnlsCremyc constructs harboring a T7 polymerase promoter. CPP sequences were inserted in frame by oligonucleotide insertion in the unique restriction sites SpeÉ and KpnÉ localized between polyhistidine and Cre recombinase coding sequences (see Additional file 1). Eukaryotic expression plasmids used in this work were designed by subcloning of the sequences encoding for CPPs-Cre-myc (without His tag) derived from pTH vectors into pTL plasmids (pSG5 derivative), in which the expression is under control of a viral SV40 promoter. All plasmid sequences were verified by sequencing. HisCreTAT expression plasmid [10] was kindly provided by Dr S.F. Dowdy (UCSD, La Jolla, CA, USA).

### Expression and purification of recombinant fusion proteins

pTH plasmids encoding different CPPs-Cre fusion proteins were used to transform the *E. coli *strain BL21(DE3)pLysS (Novagen), allowing isopropyl β-D-thiogalactoside (IPTG)-inducible expression of His-tagged proteins. An enriched medium of Luria Broth/2%glucose containing 50 μg/ml ampicillin and 34 μg/ml chloramphenicol was used for overnight preculture. 500 ml cultures of LB at 50 μg/ml ampicillin and 0.5% glucose were then inoculated by 1:20 dilution of preculture and grown at 37°C until OD_(600 nm) _reached 0.4. Protein overexpression was induced by 1 mM IPTG for 3 hours at 28°C. Cells were harvested by centrifugation and pellets were frozen dry at -80°C for storage. Cell pellets were resuspended in lysis buffer (500 mM NaCl, 25 mM Hepes, 5 mM imidazole, 5 mM MgCl_2_, 50 μg/ml DNAse, 2 μg/ml RNAse, and proteases inhibitors; Invitrogen) in a final volume of 10 ml. Cleared lysates were obtained by French press bacterial disruption and subsequent centrifugation for 1 hour at 12000 g at 4°C. After 0.45 μm filtration, the supernatant was loaded onto Histrap Ni^2+ ^affinity column (Amersham) and subsequently washed with 10 bed volumes of a 500 mM NaCl, 25 mM Hepes, 10 mM imidazole solution. Recombinant proteins were purified through one-step FPLC chromatography, by linear gradient-mediated elution from 10 to 500 mM imidazole. Fractions were analyzed by SDS-PAGE and those which showed the presence of purified protein were pooled (1 to 4 mg) and dialyzed overnight at 4°C in Pierce 10 K dialysis cassettes against pH 5 dialysis buffer (20 mM Sodium Acetate pH 5, 10 mM β-mercapto-ethanol, 40 mM NaCl). After dialysis and removal of precipitated material, protein concentration was determined using Bradford assay and 12% SDS-PAGE analysis and ranges from 0.4 to 1 μg/μl (compared to 1 to 2 μg/μl before dialysis). Proteins were stored at -80°C. Glycerol cryoprotection was omitted in order to avoid possible interference with membrane permeability upon protein exposure to live cells.

### Cell-free assay of CPP-Cre recombinase activity

Recombination assays were performed in 10 μl of buffer (50 mM Tris-HCl pH 7.5; 33 mM NaCl; 10 mM MgCl_2_) containing 250 ng of XmnÉ-linearized pLox2 plasmid (Clonetech, [47]). To compare the efficiency of recombination in vitro, a range of 100, 50, 25, 12.5 and 6.25 ng of purified Cre fusion proteins, were added, for 1/10th of the reaction volume. After 30 mn incubation at 37°C, reactions were stopped by adding 2 μl of 6× loading buffer (0.025% Bromophenol blue; 0.025% xylene cyanol; 60% glycerol; 1% SDS; 100 mM EDTA) and inactivation for 10 mn at 70°C. The reactions were then resolved on a 1.5% agarose gel. Quantification of the recombination activity was based on the comparison with a commercial Cre enzyme (NEB) and expressed according to the manufacturer unit definition.

### Protein transduction: Cellular-based assays of CPP-Cre activity

Adherent CV1B fibroblasts were kindly provided by Dr F. Tronche and used as described in the original publication [32]. In this reporter cell line, a floxed neomycin resistance/poly-adenylation cassette has to be excised to allow *E. coli lacZ *expression. Cre recombinase activity in the nucleus thus results in β-Galactosidase activity. The cells were cultured in Dulbecco's Modified Eagles medium (DMEM)/F12 medium (Gibco) supplemented with 10% fetal calf serum (FCS), penicilline (50 U/ml), streptamycine (50 μg/ml), G418 (100 μg/ml) and DnaseI (10 μg/ml). For internalization experiments, 5000 cells/well were seeded into 96-well plates coated with 1.5 μg/ml poly-ornithine (Sigma), then washed in DMEM/F12 serum-free medium. Upon internalization, cells were incubated in presence of CPP-Cre proteins diluted in DMEM/F12 supplemented with 10 mM HEPES and 100 μg/ml bovine serum albumine (BSA). Protein solutions were prepared extra-temporaneously. After protein exposure (15 to 60 mn), cells were washed with 10% FCS medium, then re-incubated for 24 or 48 hours, and subsequently monitored for reporter β-Galactosidase activity using either CPRG assay (see below) or Xgal staining (as previously described [34,35]).

### Plasmid DNA transfection experiments

CV1 fibroblasts were grown for 24 h in 10%FCS. Lipofection of 50000 cells was carried out by adding into each 24-well a mixture of Lipofectamine (Invitrogen), 200 ng of control Alkaline Phosphatase (AP)-encoding plasmid and 200 ng of CPP-Cre encoding plasmid – all diluted in Optimem medium. After 6 h of incubation, transfection medium was substituted by 10% FCS and incubation was resumed for 24 h. Cells were washed with PBS, fixed in 2.5% glutaraldehyde for 5 mn on ice and assayed for β-Galactosidase activity by Xgal staining. AP was detected using NBT and BCiP for 30 mn at room temperature.

### Enzymatic assays

To detect β-Galactosidase activity by CPRG assay, cells were washed twice with PBS, lysed in 50 μl buffer (0.1% Triton X-100, 250 mM Tris-HCl pH 8.0) frozen-thawed twice at -80°C from 37°C. 10 μl aliquots were reserved for quantitation with Bradford assay. The lysates were mixed with 150 μl of CPRG detection solution (2.5 ml CPRG substrate stock at 4 mg/ml, 35 μl β-Mercapto-ethanol 14.3 M, 7.5 ml Buffer pH 7.0 (60 mM Na_2_HPO_4_, 40 mM NaH_2_PO_4_, 10 mM KCl, 1 mM MgSO_4_). After incubation for 15, 30, 60, 90, and 120 mn, OD_(570 nm) _was assessed using an ELISA plate reader. CPRG data obtained from the wells treated with CPPs-Cre proteins were normalized against background signals obtained with vehicle-treated cells. Bradford assay was performed according to the manufacturer's instructions (Biorad). In parallel, an aliquote of each sample was used to monitor cell viability using the MTT (3-(4,5-dimethylthiazol-2-yl)-2,5-diphenyltetrazolium bromide) cell death assay (Millipore procedure). CPRG/MTT ratios were used to quantify β-Galactosidase activity.

### Immunohistochemistry

All immunodetections were performed as previously described [34-36], using the following primary antibodies at 1/500: anti-β-Galactosidase (Cappel), anti-GFP (which recognizes all GFP-derived isoforms, including YFP; Molecular Probes), and secondary antibodies from Jackson labs at 1/200 (against rabbit and mouse IgGs) conjugated to either horseradish peroxidase or fluorescein. Anti-Cre antibody raised against a Cre fusion protein was a gift from Dr A. Prochiantz.

### Cre-dependent reporter transgenic mouse strains

For most reporters in this work, the internalization of the Cre was monitored by genomic excision of a "floxed" cassette, allowing the expression of various indicator genes. The transgenic constructs contained stop or βgeo (β-Galactosidase/neomycine resistance) cassettes, flanked by two loxP sites in direct orientation. The cassette sequence is removed by excisive Cre-mediated recombination, allowing the expression of the reporter gene. Mice were genotyped using procedures from original descriptions and handled according to institutional recommendations. Excision of the underlined sequence led to the expression of the reporter moiety indicated in bold:

R26R [48]: pROSA trapped promoter – loxP – STOP – loxP – **β-geo **(**nuclear β-Galactosidase**);

RYFP [49]: pROSA trapped promoter – loxP – STOP – loxP – **YFP **(**cytoplasmic+nuclear Yellow Fluorescent Protein**);

Z/AP [50]: pCMV-pβ-actin – loxP – βgeo – loxP – **hPLAP **(**membrane human Placental Alkaline Phosphatase**);

TGZ [51,52]: pCMV – loxP – STOP – loxP – **MARCKS::eGFP**-IRES-**nlsLacZ **(**axonal Green Fluorescent Protein **and **nuclear β-Galactosidase**);

*Dbx1*-DTA [38]: *pDbx1 *– loxP – STOP – loxP – **DTa **(**Diphteric Toxin A-fragment**).

Absence of leaky transgene expression and presence of proper specific reporter expression was genetically controlled using mating with Cre-driver strains (not shown). Ubiquitous reporter expression is displayed upon recombination of the R26R, RYFP and Z/AP transgenes. In contrast TGZ expression is restricted to neuronal cells, and *Dbx1*-Dta is specific of *Dbx1*+ interneurons.

### Explant transduction procedure

Mice were handled according to institutional guidelines. Embryos were obtained from mating between syngenic mice, with detection of the vaginal plug considered as embryonic day 0.5 ('E0.5'). Pregnant mice were subjected to Nembutal overdose, and embryos transferred in PBS for dissection. Vitelline yolk sac fragments were used for genotyping according to original description of transgenic strains. As illustrated (see Additional file 3), fragments from several regions of the CNS including telencephalon, cerebellum and spinal cord were dissected from transgenic embryos or adults. Meninges were removed and CNS tissues were cultured on a polyester porous membrane (0.22 μm, Millipore), floating above serum-free medium (DMEM-F12 supplemented with Gibco N2 and B27 additives), as previously described [34-36,53]. They were challenged to express Cre-dependant transgenes upon exposure to the same sets of CPP-Cre fusion proteins, in individual wells. Vehicle solution and each conjugate were applied to at least two explants per series. Necrotic explants were discarded. Exposure was performed only 24 h after dissection to avoid non-specific internalization through membranes wounds due to dissection. Two modalities of exposure were used. In drop assay, a single 1 μl drop of purified protein in dialysis buffer was slowly laid onto the exposed surface of the explant, in the centre of the explant (see Additional file 3). In bead assay, neutral Affi-Gel beads (150–300 μm mesh, Biorad) were incubated with protein batches diluted to 400 ng/μl in PBS and rinsed, then deposited onto various spots of the explant surface. In contrast with cell culture, proteins or beads were not washed-out from the explants and transgene expression was monitored 1 to 14 days after treatment using dedicated procedures for the detection of β-Galactosidase protein or activity, human Placental Alkaline Phosphatase activity or fluorescent proteins.

## Competing interests

The authors declare that they have no competing interests.

## Authors' contributions

Project conception and experimental design: AJ, YG, LT, ED. Tools, data production and analysis: AJ, YG, LT, ED, GL. Manuscript redaction: YG, AJ, GL. All authors have read and approved the final manuscript.

## Supplementary Material

Additional file 1 **Fusion to CPPs preserved Cre recombinase activity in both cell-free and cellular context**. (A) Structure and SDS-PAGE electrophoretic profile using Sypro staining of the bacterially-produced fusion proteins, collectively designated as 'CPP-Cre' in this report. Peptidic additions did not significantly affect the apparent Mr of the Cre recombinase. The indicated conjugates averaged 45 kDa. (B) Comparison of the activity of three Cre fusion proteins by cell-free *in vitro *recombination. The Cre-mediated excision of a *loxP*-flanked target sequence reduced the size of the linear target plasmid pLox2, which released a shorter linear fragment and a circular fragment (annotated on the side of the gel). Linear pLox2 (pLox2*) was incubated for 30 mn with increasing amounts of indicated CPP-Cre proteins. Electrophoretic profiles showed equivalent recombination activities among HCT, H3C and HC, which were slightly lower than that of HNC. Underlined concentrations indicate the lowest titer with significant recombination. Note that while the unvectorized Cre displayed detectable and maximal activity at 50 ng/μl, the CPP-Cre displayed significant activity at 100 ng/μl. Click here for file

Additional file 2 **Cre reporter activity in CV1 cells upon plasmidic transfection and proteic transduction by CPP-Cre constructs**. Expression of the constructs encoding the fusion proteins resulted in nuclear accumulation of the Cre conjugates (A), and nuclear β-Galactosidase reporter expression (B) in CV1B reporter cells. Eukaryotic versions of the plasmidic DNAs were used to transfect CV1B cells using lipofectamine, extensive wash and 24 h reincubation before carrier and reporter analysis. Alongside, a transfection indicator human Placental Alkaline Phosphatase (hPLAP) encoding plasmid was co-transfected. In A, Cre (bright green staining) was immunolocalized in the nucleus of transfected cells (light blue staining). In B, β-Galactosidase activity (strong blue staining) was detected in the nucleus of transfected cells (light purple staining). Note the two sister cells derived from a single transfected parental cell. (C) Quantification of reporter-expressing cells (β-Galactosidase activity) among transfected cells (Alkaline Phosphatase activity) upon transfection by eukaryotic versions of CPP-Cre encoding plasmids. As illustrated for the three indicated proteins, equivalent levels of recombination were reached by the endogenously expressed conjugates. (D-E) CV1 cell protein transduction assays: (D) CPRG quantification of reporter-expressing cells (β-Galactosidase activity) upon exposure to 4 μM H3C or H3NC indicates similar transduction efficiency of both conjugates, suggesting that the nuclear localization signal neither improves nor impedes penetratin-mediated Cre delivery. (E) CPRG quantification of reporter-expressing cells exposed to 4 μM H3NC for either 15 or 60 mn indicates that uptake CPP-Cre conjugate is a fast process. Click here for file

Additional file 3 **Explant preparation and exposure to protein conjugates**. A) Lateral view of the brain removed from the head of an E13.5 embryo, to dissect out explants from the telencephalon (Tel) or spinal cord (Sc), in the areas delineated in red. B) Dorsal view of an adult brain, used to dissect out explants from the cerebral cortex (Ctx) or cerebellum (Cb), in the areas delineated in red. C-D) View of telencephalic (C) and spinal cord (D) explants laying flat on the membrane filter. The asterisks represent the center of the 1 μl drop of protein conjugate or vehicle upon deposition at the center of the explants, illustrated by the bottom left corner inset (size-matched 1 μl drop of ink deposited onto a plastic ruler and photographed immediately upon surface contact). Note that as the explants pump liquid from the culture medium, they create a thin liquid film at the interface with the air and surrounding the tissue – the solution rapidly diffuses through this film and covers the whole explant surface. E) Same explant as represented in Fig. 2D showing the dispersion of the transgene expressing cells in the tangential dimension. The asterisk indicates the centre of the explant at the time of drop deposition. For the whole figure, the scale bar in A represents: A, 1 mm; in B, C, 5.7 mm; in D, 500 μm; in E, 580 μm. Click here for file

Additional file 4 **Lack of transgene expression in Cre-reporter explants from different strains upon exposure to vehicle solution**. (A-D) Detection of reporter gene expression within explants upon Cre transduction in similar conditions to those illustrated in the Fig. 2 drop assay. Vehicle-treated explant lack recombinant positive cells (A, B, D: R26R; C: *Dbx1-DTA*). (E, F) Vehicle-soaked beads were applied to an explant from the TGZ strain. Neither β-Galactosidase positive nor GFP immunopositive cells could be detected (F, higher magnification around the bead implantation site). For the whole figure, the scale bar in A represents: A, 400 μm; B, D, 200 μm; C, 570 μm; E, 400 μM; F, 700 μm. Click here for file

Additional file 5 **CPP-Cre conjugates induced similar recombination levels in drop transduction assays**. Semi-quantification of the density of reporter-expressing cells in explants from four transgenic lines upon exposure to a 1 μl drop of indicated conjugates. 'Activity' is an index of mean recombination efficiency. Levels were documented by ascribing a qualitative appreciation of the amount of reporter induction, and counting the number of explants in each category: - or +: indicates none or only a few transgene-expressing cells could be detected (as in Fig. 3A and Additional file 6A, B); ++: indicates small positive cell clusters – either central or at the peripheral border (as in Fig. 2A, C and Additional file 6); +++ or ++++: describes high density of positive cells within clusters or widespread dispersal throughout the explant (e.g. Fig. 2B, 3B–C and Additional file 6E–H). Explants exposed to vehicle solution did not display induction of reporter activity, as illustrated in Additional file 4. The native Cre, tested with the RYFP strain, failed to induce significant levels of recombination. With the other conjugates (HNC, H3C, H3NC, HCT, H23C, H23NC), no significant difference could be detected, as both dense clusters and widespread dispersal of positive cells were observed. Click here for file

Additional file 6 **Dose-response of reporter activity in R26R explants upon exposure to increasing amounts of CPP-Cre conjugate**. Dose-response to 10 (A),50 (B), 100 (C, D), 200 (E, F) and 400 ng (G, H) of a 1 μl drop (asterisk) of CPP-Cre shows an almost all-or-nothing response with barely detectable x-gal positive cells under 100 ng/μl. Arrows point to representative positive cell nuclei within recombinant cell clusters. For the whole figure, the scale bar in A represents: A, 140 μm; B, D, 120 μm; C, E, G, 350 μm; D, 50 μm; F, 100 μm; H, 80 μm. Click here for file
