# Supplementary figures and images for: Efficient CPP-mediated Cre protein delivery to developing and adult CNS tissues
Source: BMC Biotechnol. 2009 Apr 24;9:40. doi: 10.1186/1472-6750-9-40 (PMC2680837; doi:10.1186/1472-6750-9-40)

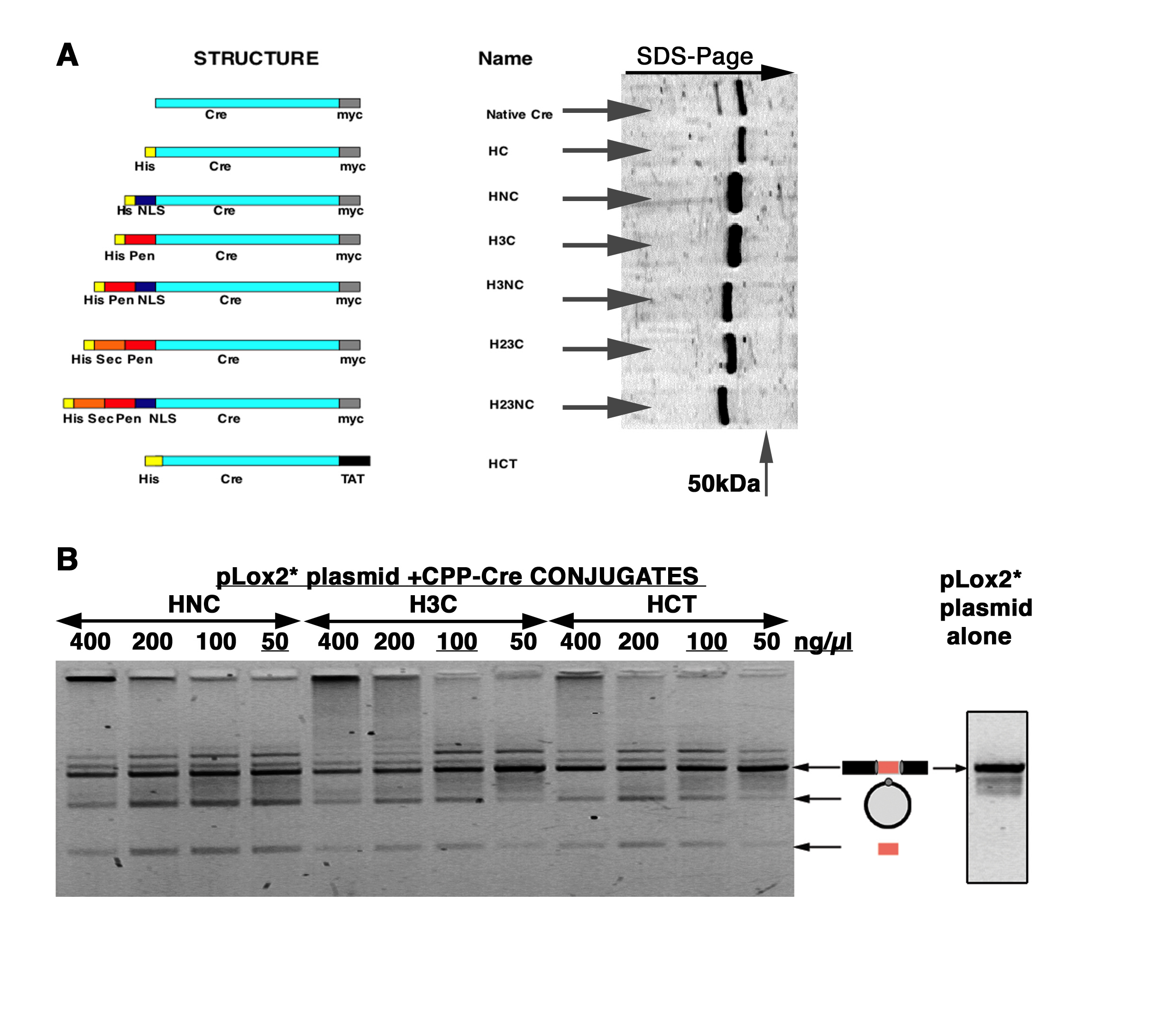

Supplement: Additional file 1 — Fusion to CPPs preserved Cre recombinase activity in both cell-free and cellular context. (A) Structure and SDS-PAGE electrophoretic profile using Sypro staining of the bacterially-produced fusion proteins, collectively designated as 'CPP-Cre' in this report. Peptidic additions did not significantly affect the apparent Mr of the Cre recombinase. The indicated conjugates averaged 45 kDa. (B) Comparison of the activity of three Cre fusion proteins by cell-free in vitro recombination. The Cre-mediated excision of a loxP-flanked target sequence reduced the size of the linear target plasmid pLox2, which released a shorter linear fragment and a circular fragment (annotated on the side of the gel). Linear pLox2 (pLox2*) was incubated for 30 mn with increasing amounts of indicated CPP-Cre proteins. Electrophoretic profiles showed equivalent recombination activities among HCT, H3C and HC, which were slightly lower than that of HNC. Underlined concentrations indicate the lowest titer with significant recombination. Note that while the unvectorized Cre displayed detectable and maximal activity at 50 ng/μl, the CPP-Cre displayed significant activity at 100 ng/μl. [file 1472-6750-9-40-S1.jpeg]

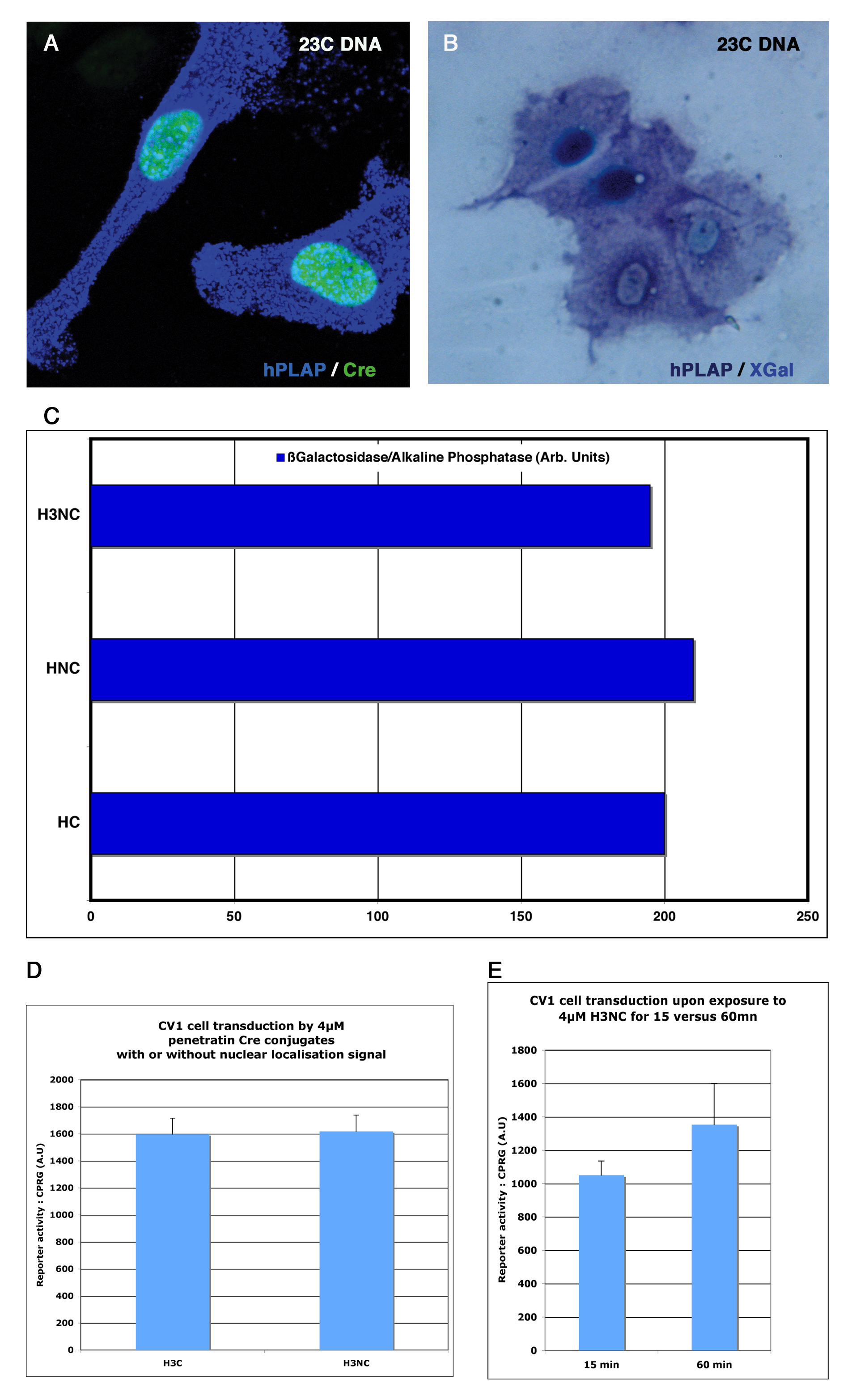

Supplement: Additional file 2 — Cre reporter activity in CV1 cells upon plasmidic transfection and proteic transduction by CPP-Cre constructs. Expression of the constructs encoding the fusion proteins resulted in nuclear accumulation of the Cre conjugates (A), and nuclear β-Galactosidase reporter expression (B) in CV1B reporter cells. Eukaryotic versions of the plasmidic DNAs were used to transfect CV1B cells using lipofectamine, extensive wash and 24 h reincubation before carrier and reporter analysis. Alongside, a transfection indicator human Placental Alkaline Phosphatase (hPLAP) encoding plasmid was co-transfected. In A, Cre (bright green staining) was immunolocalized in the nucleus of transfected cells (light blue staining). In B, β-Galactosidase activity (strong blue staining) was detected in the nucleus of transfected cells (light purple staining). Note the two sister cells derived from a single transfected parental cell. (C) Quantification of reporter-expressing cells (β-Galactosidase activity) among transfected cells (Alkaline Phosphatase activity) upon transfection by eukaryotic versions of CPP-Cre encoding plasmids. As illustrated for the three indicated proteins, equivalent levels of recombination were reached by the endogenously expressed conjugates. (D-E) CV1 cell protein transduction assays: (D) CPRG quantification of reporter-expressing cells (β-Galactosidase activity) upon exposure to 4 μM H3C or H3NC indicates similar transduction efficiency of both conjugates, suggesting that the nuclear localization signal neither improves nor impedes penetratin-mediated Cre delivery. (E) CPRG quantification of reporter-expressing cells exposed to 4 μM H3NC for either 15 or 60 mn indicates that uptake CPP-Cre conjugate is a fast process. [file 1472-6750-9-40-S2.jpeg]

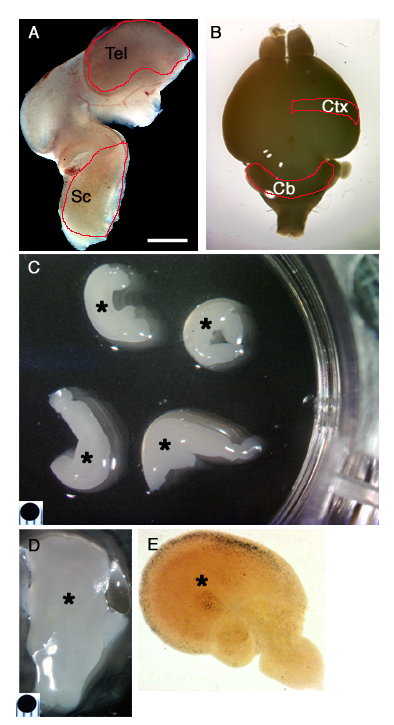

Supplement: Additional file 3 — Explant preparation and exposure to protein conjugates. A) Lateral view of the brain removed from the head of an E13.5 embryo, to dissect out explants from the telencephalon (Tel) or spinal cord (Sc), in the areas delineated in red. B) Dorsal view of an adult brain, used to dissect out explants from the cerebral cortex (Ctx) or cerebellum (Cb), in the areas delineated in red. C-D) View of telencephalic (C) and spinal cord (D) explants laying flat on the membrane filter. The asterisks represent the center of the 1 μl drop of protein conjugate or vehicle upon deposition at the center of the explants, illustrated by the bottom left corner inset (size-matched 1 μl drop of ink deposited onto a plastic ruler and photographed immediately upon surface contact). Note that as the explants pump liquid from the culture medium, they create a thin liquid film at the interface with the air and surrounding the tissue – the solution rapidly diffuses through this film and covers the whole explant surface. E) Same explant as represented in Fig. 2D showing the dispersion of the transgene expressing cells in the tangential dimension. The asterisk indicates the centre of the explant at the time of drop deposition. For the whole figure, the scale bar in A represents: A, 1 mm; in B, C, 5.7 mm; in D, 500 μm; in E, 580 μm. [file 1472-6750-9-40-S3.jpeg]

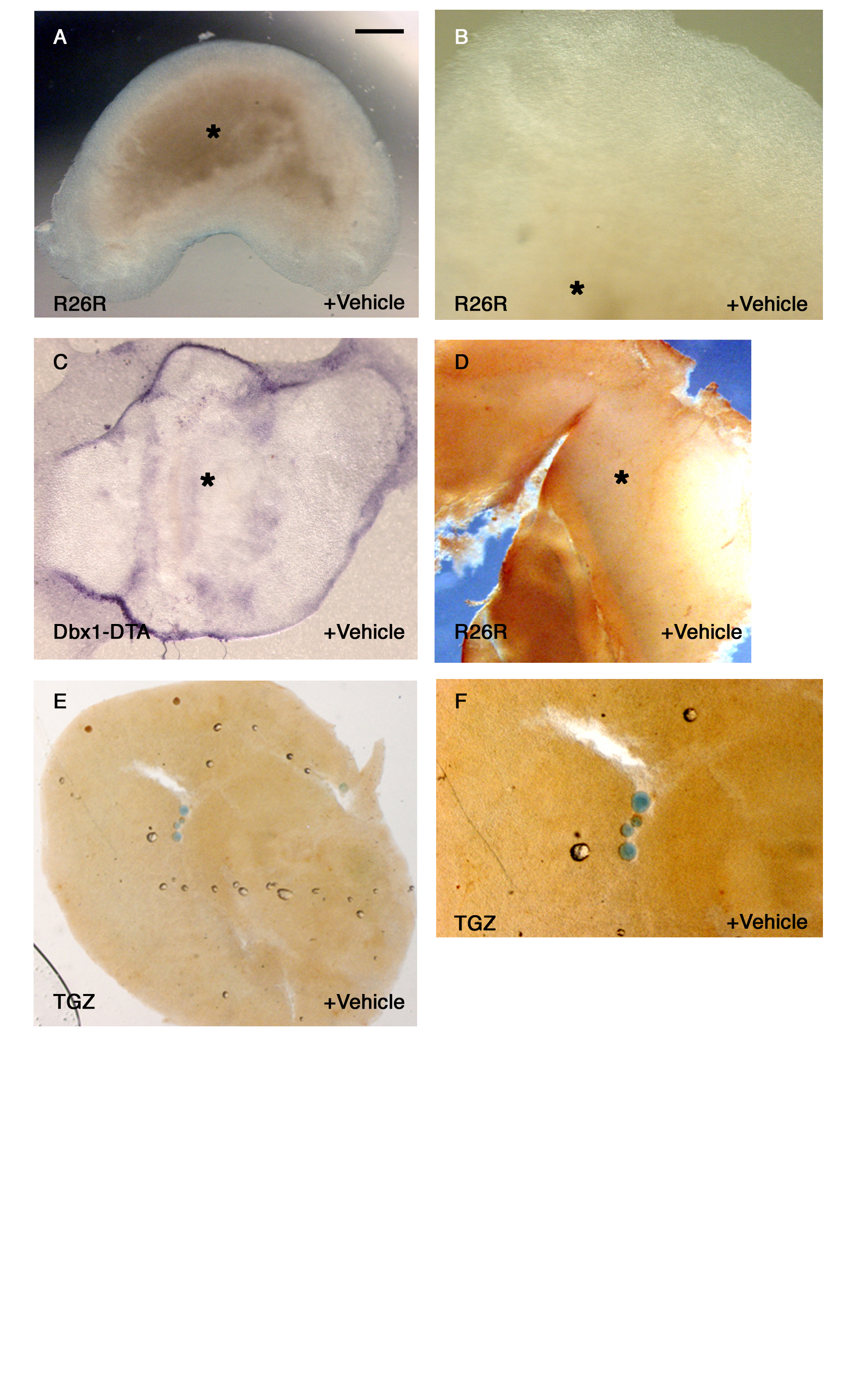

Supplement: Additional file 4 — Lack of transgene expression in Cre-reporter explants from different strains upon exposure to vehicle solution. (A-D) Detection of reporter gene expression within explants upon Cre transduction in similar conditions to those illustrated in the Fig. 2 drop assay. Vehicle-treated explant lack recombinant positive cells (A, B, D: R26R; C: Dbx1-DTA). (E, F) Vehicle-soaked beads were applied to an explant from the TGZ strain. Neither β-Galactosidase positive nor GFP immunopositive cells could be detected (F, higher magnification around the bead implantation site). For the whole figure, the scale bar in A represents: A, 400 μm; B, D, 200 μm; C, 570 μm; E, 400 μM; F, 700 μm. [file 1472-6750-9-40-S4.jpeg]

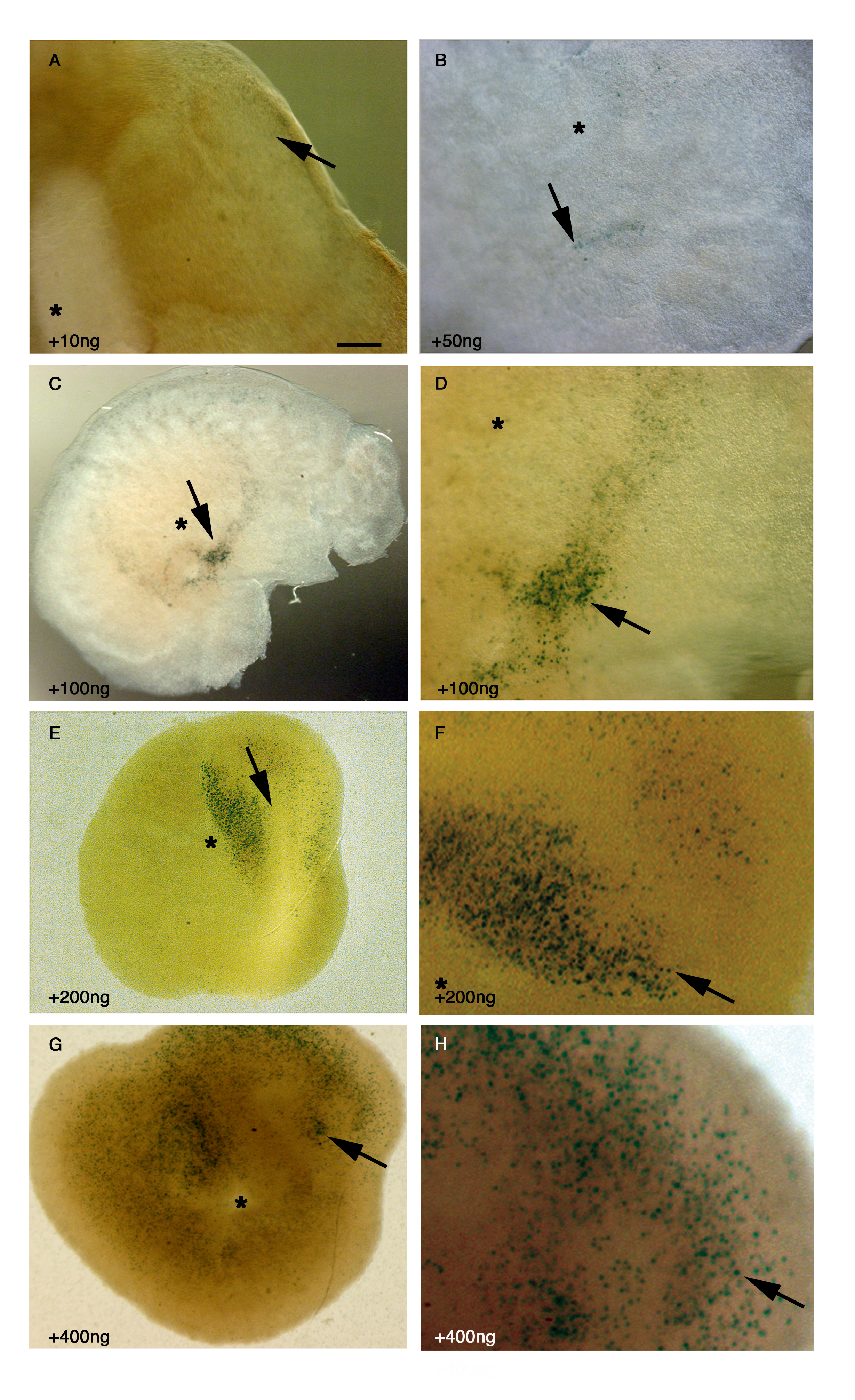

Supplement: Additional file 6 — Dose-response of reporter activity in R26R explants upon exposure to increasing amounts of CPP-Cre conjugate. Dose-response to 10 (A),50 (B), 100 (C, D), 200 (E, F) and 400 ng (G, H) of a 1 μl drop (asterisk) of CPP-Cre shows an almost all-or-nothing response with barely detectable x-gal positive cells under 100 ng/μl. Arrows point to representative positive cell nuclei within recombinant cell clusters. For the whole figure, the scale bar in A represents: A, 140 μm; B, D, 120 μm; C, E, G, 350 μm; D, 50 μm; F, 100 μm; H, 80 μm. [file 1472-6750-9-40-S6.jpeg]
