# Supplementary material for: Efficient CPP-mediated Cre protein delivery to developing and adult CNS tissues
Source: BMC Biotechnol. 2009 Apr 24;9:40. doi: 10.1186/1472-6750-9-40 (PMC2680837; doi:10.1186/1472-6750-9-40)
Supplement: Additional file 5 — CPP-Cre conjugates induced similar recombination levels in drop transduction assays. Semi-quantification of the density of reporter-expressing cells in explants from four transgenic lines upon exposure to a 1 μl drop of indicated conjugates. 'Activity' is an index of mean recombination efficiency. Levels were documented by ascribing a qualitative appreciation of the amount of reporter induction, and counting the number of explants in each category: - or +: indicates none or only a few transgene-expressing cells could be detected (as in Fig. 3A and Additional file 6A, B); ++: indicates small positive cell clusters – either central or at the peripheral border (as in Fig. 2A, C and Additional file 6); +++ or ++++: describes high density of positive cells within clusters or widespread dispersal throughout the explant (e.g. Fig. 2B, 3B–C and Additional file 6E–H). Explants exposed to vehicle solution did not display induction of reporter activity, as illustrated in Additional file 4. The native Cre, tested with the RYFP strain, failed to induce significant levels of recombination. With the other conjugates (HNC, H3C, H3NC, HCT, H23C, H23NC), no significant difference could be detected, as both dense clusters and widespread dispersal of positive cells were observed. [file 1472-6750-9-40-S5.doc]

| **Reporter** | **Detection** | **Age** | **N** | **Conjugate**  **(3,23,TAT)** | **Titer (ng/µl)** | **Activity** |
| --- | --- | --- | --- | --- | --- | --- |
| **R26R** | X-Gal | E14.5 | 16 | H3C  HNC | 100  100 | **+++**  **++** |
|  | X-Gal | E15.5 | 16 | H3C  HNC | 100  100 | **++++**  **+** |
|  | X-Gal or IC_ßGal | Adult | 20 | All CPP  C or HNC | 100  100 | **+++**  **-** |
| **RYFP** | Live FITC | E14.5 | 16 | H3C  HNC | 200  200 | **++**  **++** |
|  | IC_GFP | E15.5 | 14 | H3C  HNC | 100  100 | **+++**  **+++** |
|  | IC_GFP | Adult | 12 | H3C  HNC | 100  100 | **+**  **-** |
| **TGZ** | IC_GFP | E14.5 | 16 | All CPP | 100 | **+++** |
|  |  | Adult | 18 | HNC | 200 | **-** |
| **Dbx1-DTA** | IC_Casp3^Act^ | E9.5  E14.5 | 8  8 | H3C  All CPP | 100  200 | **++++**  **++++** |
